# In-Situ Synchrotron X-ray Study on the Structure Variation of Morphology-Identified Injection-Molded β-Nucleated iPP under Tensile Deformation

**DOI:** 10.3390/polym13213730

**Published:** 2021-10-28

**Authors:** Jianhong Chen, Konrad Schneider, Gert Heinrich

**Affiliations:** 1 Fujian Provincial Key Laboratory of Functional Materials and Applications, Xiamen University of Technology, Ligong Road 600, Xiamen 361024, China; 2 Leibniz-Institut für Polymerforschung Dresden e.V., Hohe Str. 6, 01069 Dresden, Germany; gheinrich@ipfdd.de; 3 Institut für Textilmaschinen und Textile Hochleistungswerkstofftechnik, Technische Universität Dresden, Zellescher Weg 17, 01062 Dresden, Germany

**Keywords:** β-nucleated polypropylene, morphological distribution, deformation, structure variation, synchrotron X-ray

## Abstract

The deformation behavior of semi-crystalline polymers is strongly dependent on the morphology formed during processing. In this study, in-situ synchrotron X-ray was firstly used to identify the morphological distributions of injection-molded isotactic polypropylene (iPP) with different concentrations of β-nucleating agent. It was found that under relatively high concentration of β-nucleating agent (i.e., ≥0.03 wt.%), the outer region (skin and shear region) of the iPP was dominated by mainly highly oriented α-phase as well as certain amount γ-phase, while the core region was rich in β-crystals with little if any orientation. The addition of the β-nucleating agent was beneficial for the formation of lamellae with large lamellar stacking distance in the shear layer. Then the synchrotron X-ray was applied to study the structure variation of those morphology-identified samples under tensile deformation. It was found that voids and cavities along the stretching direction existed in the deformed iPP samples and their volume increased with increasing concentration of β-nucleating agent. The increased volume of void and cavity was associated with the β to α phase transition, which mainly occurred at the core region. In addition, upon stretching crystalline fragmentation and rearrangement took place following the formation of thinner lamellae.

## 1. Introduction

Isotactic polypropylene (iPP), as one of the most utilized thermoplastic polymers, has been identified as a polymorphic material including mainly monoclinic (α), pseudo-hexagonal (β) and orthorhombic (γ) types. The α type is thermodynamically stable and is by far most common [1]. The β type has been termed as metastable one with high degree of disorder and can be fabricated by crystallizing (i) in a temperature gradient, (ii) under strong shear and (iii) through epitaxial growth on the specific heterogeneous nucleating agents. The γ-iPP is relatively rare, however, it is favored when iPP crystallizes under elevated pressure or the isotactic sequence length is interrupted [2,3,4,5]. Different crystal forms may endow iPP products with significantly varied properties. α-iPP usually exhibits good mechanical strength, however, poor impact toughness at low temperatures. Compared to α-iPP, the β-iPP has a low E-modulus and yield stress, but high elongation at break and ultimate tensile strength, which may be attributed to the β to α transition taking place during the necking, resulting in the formation of α-phase with enhanced strength. The impact strength and toughness of β-iPP are also superior to α-iPP [6,7]. Up to now, the most effective and convenient way to obtain iPP with high β-content is adding certain β-nucleating agent.

In common industrial production, iPP plastic is usually shaped by various processing operations, such as injection molding, extrusion, fiber spinning, etc., and the molten iPP is often exposed to complicated flow fields and temperature gradient, which strongly affect its crystallization kinetics and final morphology [8,9,10,11,12]. Taking injection molding as an example, 3 distinct layers exist in the iPP sheet, i.e., a highly oriented nonspherulitic skin, a shear-nucleated intermediate layer, and a core layer [13,14]. The very thin skin layer is mainly composed of amorphous phase due to the rapid cooling, while the core layer consists predominantly of spherulitic structure associated with the experienced low cooling and shear rate, which allow for a good relaxation of molecular chains. The shear zone or shear layer, which undergoes extremely high shear rate, separates the skin and core layer, and it is the most important layer determining the shear-induced properties. The introduction of β-nucleating agent would further complicate the crystallization process and change the morphology of the layers. It is known that the addition of β-nucleating agent may lead to the competitive growth of α- and β-crystals under flow field. The increase of shear rate would result in the decrease of β-crystallinity of sample [15,16,17,18]. However, the morphology changes in the above-described three layers as a function of β-nucleating agent concentration have not been studied and described in detail yet [3,19].

During stretching deformation of semi-crystalline polymers with various crystalline structures, crystal transition between different structures often occurs. For example, during drawing of nylon 6 fibers an apparent transition from γ-phase crystals to α-phase crystals takes place, which is explained by that the γ-phase crystals were destroyed during the drawing process and then converted into fiber structure of the α-phase form [20]. Such strain-induced phase transition has also been widely reported in poly(ω-pentadecalactone) (PPDL), poly(l-lactic acid) (PLA), iPP and polyethylene (PE) [21,22,23,24,25,26,27,28,29,30]. During tensile drawing, the deformation-induced cavitation also plays a crucial role in the structure transformation of polymers [24,31,32,33,34]. The cavitation often appears at suitable stretching conditions and is visible as whitening at macroscopic scale during tensile deformation of polymers. The formation and growth of cavities are primarily responsible for the volume change during tensile drawing [35]. Pawlak et al. reported that there was a competition between cavitation and activation of crystal plasticity during deformation. The cavitation occurred in polymers with crystals of higher plastic resistance, whereas the plastic deformation dominated in polymers with lower plastic resistance [31]. The appearance of cavitation generally led to a variation of mechanical properties of polymers.

Different initial structures and morphologies may also strongly influence the material properties and the structure evolution during the deformation. To better understand the deformation mechanism of β-nucleated iPP prepared by common injection molding, it is necessary to understand the morphological distributions in different layers. Conventional X-ray scattering cannot be used to investigate the morphological distribution across the thickness direction since its beam size is so big that both the shear layer and core layer are simultaneously illuminated. Thanks to the microbeam with beam size of 20 × 20 µm^2^ at MINAXS beamline, Petra III in Hamburg Germany, the beam can be precisely positioned to characterize the crystalline structures in different layers allowing us obtain the morphology-identified injection-molded samples.

In the current study, injection-molded iPP samples with different concentrations of β-nucleating agent will be firstly scanned by the synchrotron X-ray microbeam to identify their overall morphological distributions in thickness direction, including distributions of crystallinity, long period and orientation, etc. Then those morphology-identified samples will be stretched to strain of about 300%. The strain of 300% is selected, since morphology at this strain may reflect the key features of plastic deformation. The structure variation, involving the changes of crystallinity, crystal transition, orientation, and cavitation, etc., will be discussed and connected with their original morphological structure determined by the processing.

## 2. Materials and Methods

### 2.1. Materials and Sample Preparation

In this study, we used a commercial iPP homopolymer (grade HD 120 MO) manufactured by Borealis (Linz, Austria) with a melt flow index of 8 g/10 min (at 230 °C and 2.16 kg), *M*_w_ = 365 kg/mol and *M*_w_/*M*_n_ = 5.4 [36]. The β-nucleating agent, *N*,*N′*-dicyclohexyl-2,6-naphthalenedicarboxamide (NJS), was produced by Rika International Limited (Oldham, UK).

The following processing was employed to prepare the β-nucleated iPP. Firstly, iPP was melt mixed with 0.5 wt.% NJS at 220 °C to obtain a master batch through a single screw extruder (Brabender, Duisburg, Germany, L/D ratio of the screw is 25 and D = 19 mm). The master batch was then further melt compounded with iPP pellets using a co-rotating twin screw extruder (Leistritz, ZSE 27, Nürnberg, Germany, D = 27 mm and L/D = 36). In this process, a temperature program within 200–220 °C from hopper to die and a screw speed of 200 rpm were adjusted, under which a throughput of 10 kg/h was generated. After pelletizing and drying, the samples were injection molded into rectangular sheets of dimension 80 × 80 × 1 mm^3^ by an injection molding machine (Demag, Ergotech 100/420-310, Schwaig, Germany) under a barrel temperature of 225 °C and a mold temperature of 40 °C. Three β-nucleated iPP samples with the NJS concentrations of 0.01, 0.03, and 0.1 wt.% were prepared and named as Bpp01, Bpp03 and Bpp10, respectively. For comparison, neat iPP without NJS was also prepared under the same processing conditions and designated as Bpp0. It is worth noting that during the processing, the β-nucleating agent in Bpp01 was totally dissolved while in Bpp03 and Bpp10; certain insoluble β-nucleating agent particles existed due to the relatively large concentration [17,37,38].

**Figure 1:**
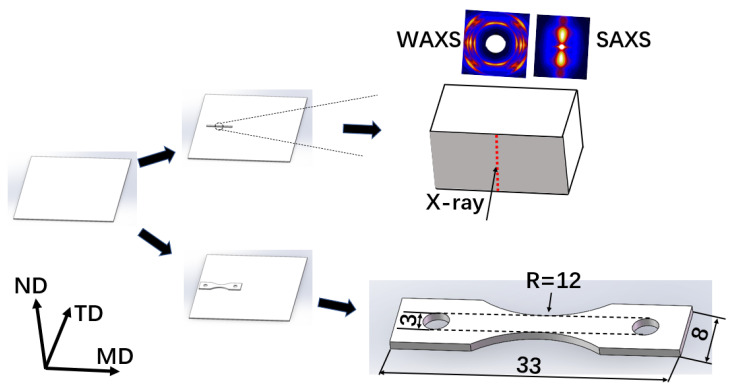
Schematics of preparation of morphology-scanned and mini-dumbbell specimens from the injection-molded iPP sheets for in-situ morphological scanning and tensile measurements, respectively. Noting that the X-ray beam is along the TD direction for the morphology-scanned specimen, while along the ND direction for the mini-dumbbell specimen.

For tensile experiment, mini-dumbbell specimens were cut from the 1 mm thick sheets by CNC milling [39]. The specimens with their length along the injection direction were taken from the position near the inlet with the distance of sample center to the inlet being about 18 mm (Figure 1). For the morphological scan experiment, specimens with dimensions of 16 × 1 × 1 mm^3^ were prepared from the 1 mm thick sheets. The specimens are located at the same position as tensile specimens with their length along the injection direction (Figure 1).

### 2.2. X-ray Measurements

The synchrotron X-ray measurements were carried out at beamline P03 of Petra III, DESY, Hamburg, Germany. The beam size was 20 × 20 µm^2^ and the wavelength was 0.1069 nm. The exposure time was 0.5 s and the sampling rate was 0.33 s^−1^. Two-dimensional WAXS and SAXS patterns were collected using two separate detectors Pilatus 300 K and Pilatus 1 M (Paul Scherrer Institute, Villigen, Switzerland), respectively, with a readout time of 3 ms. The sample to detector distance was 2600 mm for SAXS and 129.5 mm for WAXS. All of the X-ray patterns were corrected for background scattering, air scattering, and beam fluctuations.

### 2.3. Morphological Scan Measurement

The morphological scanning measurement was carried out with the primary X-ray beam along the TD direction of sample by vertically shifting the sample (Figure 1).

### 2.4. Tensile Deformation Measurement

Uniaxial tensile deformation was performed on a custom-made miniature tensile machine equipped with a heating device, which contained a porous ceramic material allowing generation of a homogenous heat convection [39]. The samples were symmetrically stretched at elevated temperature of 90 °C under stretching rate of 0.1 mm/s. A digital camera was used to in-situ follow the deformation process of the sample without hindering the X-ray path. The strain ε of the stretched sample was measured optically by observing the deformation of a grid pattern on the sample surface, with a mesh size of 0.35 mm, printed by using a self-made flexible ink. The center of the specimen, which was irradiated by X-ray beam, was left blank [40].

### 2.5. Data Evaluation for X-ray Scattering

#### 2.5.1. Overall Crystallinity Index and Individual Crystallinity Index

Linear WAXS profiles were obtained by circular integration of the 2D-WAXS patterns. Subsequently, the peaks in the linear WAXS profiles were separated via Gaussian fit by means of a self-written peak-fitting procedure in PV-wave from Visual Numerics (Texas Houston, TX, USA) [41]. The overall crystallinity index was then calculated by using the following Equation (1) [32,42]:

(1) $$X_{c}=\frac{\sum A_{cryst}}{\sum A_{cryst}+A_{amorph}}$$

where $\sum A_{cryst}$ is the total crystalline peak areas and $A_{amorph}$ is the amorphous peak area.

Certain γ-phase was found in the outer layer of the injection molding sheets and cannot be neglected in the morphological scan experiment. In this case, 3 kinds of crystal modifications usually coexisted in the samples. The relative amount of the γ-crystals *K_γ_* and β-crystals *K_β_* were estimated using the Turner-Jones method [43] in Equations (2) and (3), respectively:

(2) $$K_{\gamma }=\frac{A_{\gamma }\left(117\right)}{A_{\gamma }\left(117\right)+A_{\alpha }\left(130\right)}$$

(3) $$K_{\beta }=\frac{A_{\beta }\left(300\right)\times \left(1-K_{\gamma }\right)}{A_{\beta }\left(300\right)+\left(A_{\alpha }\left(110\right)+A_{\alpha }\left(040\right)+A_{\alpha }\left(130\right)\right)\times \left(1-K_{\gamma }\right)}$$

where *A_γ_*(117), *A_β_*(300), *A_α_*(130), *A_α_*(110) and *A_α_*(040) are the areas of the (117) reflection peak of the γ-iPP, (300) reflection peak of the β-iPP, (130), (110) and (040) reflection peaks of the α-iPP, respectively.

The individual crystallinity index of the γ-form crystals *X_γ_*, β-form crystals *X_β_* and α-form crystals *X_α_*, respectively, were then given by:

(4) $$X_{\gamma }=X_{c}\cdot K_{\gamma }$$

(5) $$X_{\beta }=X_{c}\cdot K_{\beta }$$

(6) $$X_{\alpha }=X_{c}-X_{\beta }-X_{\gamma }$$

#### 2.5.2. Crystal Size

The crystal size was calculated from the full width at half-maximum (FWHM) of the fitted crystalline peaks according to the Debye-Scherrer equation [44]:

(7) $$L_{hkl}=\frac{K\lambda }{\beta_{1/2}\cos\Theta }$$

where *L_hkl_* represents the mean crystallite size in the normal direction of the (h k l) reflection plane and *β*_1/2_ is the FWHM of the diffraction peak (h k l) in radians. The shape factor K was set as 0.9 for polymer systems [20,22,45].

#### 2.5.3. Long Period

The long period, spacing between adjacent crystalline lamellae layers, was calculated from circular averaged 1-D SAXS data by Equation (8) [25,46]:

(8) $$L_{B}=\frac{2\pi }{q_{max}}$$

where *q_max_* represents the peak position in Lorentz corrected scattering intensity plot.

#### 2.5.4. Orientation Degree

The crystalline orientation was estimated from the WAXS pattern through Hermans’ orientation function [47] defined as follows:

(9) $$f_{H}=\frac{3<cos^{2}\emptyset >-1}{2}$$

where ∅ is the angle between the normal to the (040) reflection plane and the reference axis (a direction perpendicular to the machine direction in 2D-WAXS pattern). The term <*cos*2∅> is defined as

(10) $$<cos^{2}\emptyset >=\frac{\int_{0}^{\pi /2}I\left(\theta \right)cos^{2}\left(\theta \right)sin\left(\theta \right)d\theta }{\int_{0}^{\pi /2}I\left(\theta \right)sin\left(\theta \right)d\theta }$$

with *I*(*θ*) obtained from the scattering intensity at the azimuthal angle *θ* of the (040) reflection ring in 2D-WAXS pattern. The *f_H_* = 1 means that the reflection plane is parallel to the machine direction (∅ = 0°), *f_H_* = −0.5 means that the reflection plane is perpendicular to the machine or stretching direction (∅ = 90°) and *f_H_* = 0 means that the reflection plane has no preferred orientation.

## 3. Results and Discussion

### 3.1. Morphological Distributions

**Figure 2:**
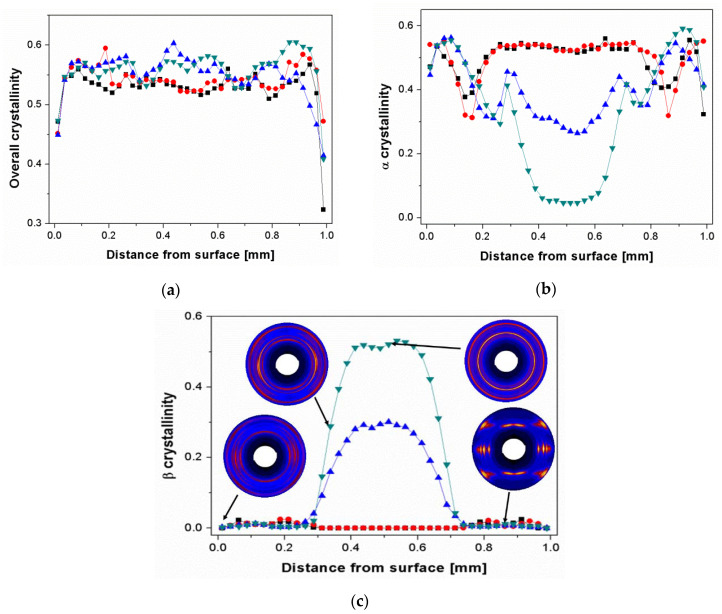

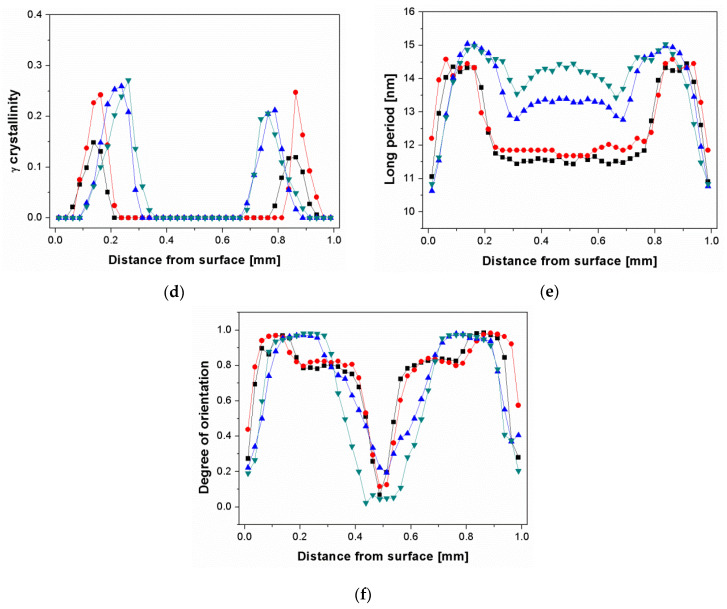
Distributions of overall crystallinity (**a**), α-crystallinity (**b**), β-crystallinity (**c**), γ-crystallinity (**d**), long period (**e**) and orientation degree (**f**) for iPP samples within an injection-molded iPP plate with different amount of nucleating agent: Bpp0 (■), Bpp01 (●), Bpp03 (▲), and Bpp10(▼). Selective 2D-WAXS patterns of Bpp10 at typical positions are shown in the inset (**c**). Notice that the core center locates at distance of 0.5 mm from surface.

The distributions of overall crystallinity, different crystalline phases, long period as well as orientation degree through the depth of the β-nucleated iPP sheets are shown in Figure 2a–g. It can be seen in Figure 2a that at the surface area all of the samples exhibited low overall crystallinity, i.e., from 0.32 to 0.47, which was attributed to the quick cooling rate restricting the formation of crystallites. Apart from the surface, the values of overall crystallinity were higher than that at surface and though fluctuated a little, remained relatively constant at around 0.53. The corresponding individual α-, β-, and γ-crystallinity distributions are presented in Figure 2b–d. It can be found that the β-crystallinity at the region between surface and 0.3 mm apart from surface was very low, even in Bpp10 with highest β-nucleating agent concentration (Figure 2c). This was reasonable since the shear rate at this region was extremely high, which induced a large amount of oriented α-crystals and thus inhibited the formation of β-crystals [15,16,17]. For Bpp0 and Bpp01 the core was solely composed of α-crystals. The absence of β-crystals in core layer of Bpp01 indicated that the few amount of β-nucleating agent did not induce the formation of β-crystals during the injection-molding process. Our previous results showed that the NJS with the same concentration of 0.01 wt.% exhibited relatively high β-nucleating ability (β crystallinity of final solid sample reached above 0.31 after shear-induced or quiescent crystallization at isothermal temperature of 138 °C for 5 min) [17]. The declined effectiveness of the NJS in Bpp01 can be associated with the injection molding processing history. Due to the relatively high barrel temperature of 225 °C, the few β-nucleating agent was totally dissolved in Bpp01 [17,37,48] and during the subsequent rapid cooling process the formation of fine crystals of NJS, which served as the precursor to induce the formation of β-crystals, may be strongly restricted, resulting in the inactivity of NJS. With increasing NJS concentration, the β-crystallinity increased at the expense of α-phase in the core layer (Bpp03 and Bpp10 in Figure 2b,c). The β-crystallinity gradually increased from around 0.3 mm apart from the surface and reached maximum at the core center, indicating that the molecular chains changed to coiled state due to the lower shear and cooling rates allowing a good relaxation of molecular chains in the core region.

In addition, the very high shear effect at the region between surface and 0.35 mm apart from the surface also led to formation of highly oriented γ-crystals (Figure 2c inset and Figure 2d). This result is consistent with results of Kalay et al., who reported that the occurrence of γ-crystals in injection moldings was associated with high molecular alignment [13]. Compared with pure iPP, the addition of β-nucleating agent resulted in a higher content of γ-form crystals in this region and made the γ-crystallites locate at the position farther to the surface. According to the results of Housmans et al., the occurrence area of γ-phase may be roughly associated with the position of shear layer [3].

The long period distributions are shown in Figure 2e. All samples exhibited a low value of long period at the surface, where fewer crystallites were formed due to fast cooling rate. The long period gradually increased to a maximum in the region around 0.1–0.2 mm apart from surface and then decreased in the region about 0.2–0.3 mm apart from surface. The long period of Bpp03 and Bpp10 were larger than that of pure iPP and Bpp01 in the highly sheared region, indicating that the presence of nucleating agent particles was beneficial for formation of lamellae with increased stacks distance. This can be explained by the idea that the particles may promote the alignment of molecular chains and induce more oriented nuclei [17,49]. The long period increased with increasing concentration of β-nucleating agent at the core region. This was reasonable since the β-nucleating agent may promote the formation of β-crystals leading to the increase of lamellar stacking distance [16]. In addition, it can be observed that the distribution of long period was relatively flat for iPP without or with little nucleating agent (Bpp0 and Bpp01) at the core region indicating that the molecular chains of α-crystals in these two samples shared similar thermomechanical history at this region. On the other hand, for iPP with high concentration of β-nucleating agent (Bpp03 and Bpp10), the distributions of long period exhibited inverted U-shape at the core region, which can be mainly attributed to the change of β-content as shown in the Figure 2c, noting that β-lamellae usually exhibited a larger long period compared with α-lamellae under the same crystallization temperature [16].

Figure 2f shows the distributions of orientation functions for iPP nucleated with various concentrations of β-nucleating agent. In all of the samples, the orientation degree increased, starting at a low value at the surface, where fewer ordered crystallites were formed due to fast cooling, to a maximum close to 1 in the region around 0.05–0.3 mm apart from surface, after which it generally decreased to a low value close to 0 in the core region. Special attention was paid to the region around 0.05–0.3 mm apart from surface, where the orientation degree was very high. Combining with the Figure 2b–d, it can be concluded that the outer region (mainly skin + shear region) in the all of samples prepared by traditional injection molding was dominated by mainly highly oriented α-crystals and certain amount of γ-crystals. However, for the core region, the samples with relatively high concentration of NJS (Bpp03 and Bpp10) were rich in β-crystals with little if any orientation, while the samples without or with few NJS (Bpp0 and Bpp01) were still mainly composed of little if any oriented α-form crystals.

### 3.2. Structure Variation under Tensile Deformation

**Figure 3:**
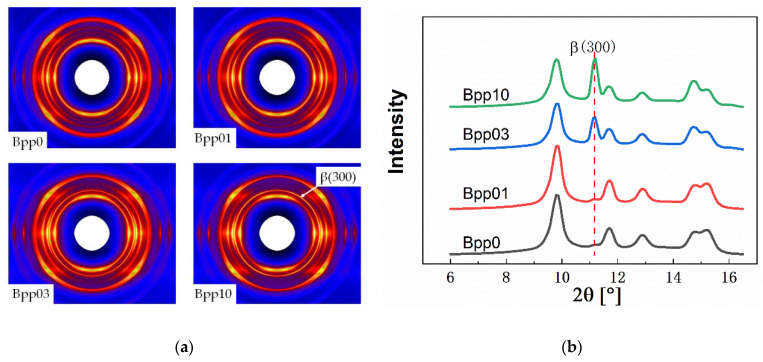
2D-WAXS patterns of different undeformed injection-molded iPP samples (**a**), note: injection direction is vertical) and the corresponding 1-D WAXS intensity profiles (**b**).

Figure 3 displays the 2D-WAXS patterns and the corresponding 1D-WAXS intensity curves of undeformed injection-molded iPP samples with different concentrations of β-nucleating agent. As can be seen, all of the samples exhibited highly oriented structure (Figure 3a). In addition, with increasing concentration of β-nucleating agent, the scattering of (300) reflection enhanced (Figure 3a,b), indicating the increase of β-crystallinity.

**Figure 4:**
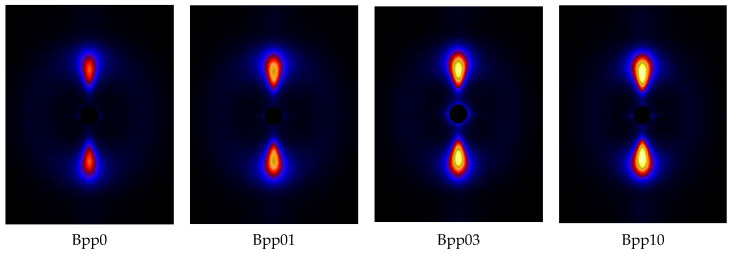
SAXS patterns for undeformed injection-molded iPP samples with different concentrations of β-nucleating agent, injection direction is vertical.

**Figure 5:**
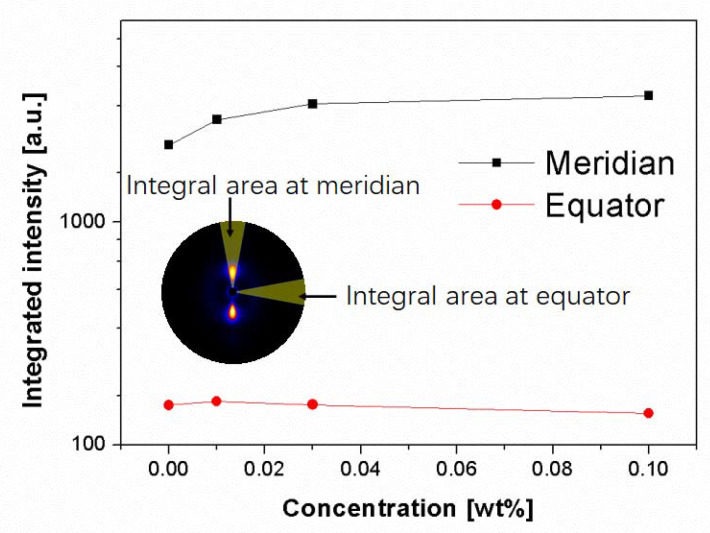
Integrated intensity along meridian and equator for undeformed injection-molded iPP as a function of β-nucleating agent concentrations. The integral region is shown in the inset.

Figure 4 displays the SAXS patterns of undeformed injection-molded iPP samples with different concentrations of β-nucleating agent and Figure 5 shows the integrated intensities in meridional and equatorial direction for the SAXS-patterns shown in Figure 4 using the following expressions:

(11) $$I_{equator}\;=\;\int_{q_{1}}^{q_{2}}\int_{-10{}^{\circ}}^{10{}^{\circ}}I\left(q,\phi \right)d\phi dq$$

and

(12) $$I_{meridian}\;=\;\int_{q_{1}}^{q_{2}}\int_{80{}^{\circ}}^{100{}^{\circ}}I\left(q,\phi \right)d\phi dq$$

where *I*(*q*, *φ*) represents the scattered intensity at angle *φ* and scattering vector *q* (here, *q*_1_ = 0.084 nm^−1^ and *q*_2_ = 1.43 nm^−1^, the corresponding integral area can be seen in the Figure 5). The SAXS scattering intensities are related to the electronic density contrast and to the volume fraction of the objects. It can be seen that the intensity at the meridian was much higher than that at equator. The intensity at equator was almost independent of the concentration of β-nucleating agent, while the intensity at meridian increased with increasing concentration of β-nucleating agent, indicating that lamellar structure became more perfect as the concentration of the nucleating agent increased.

**Figure 6:**
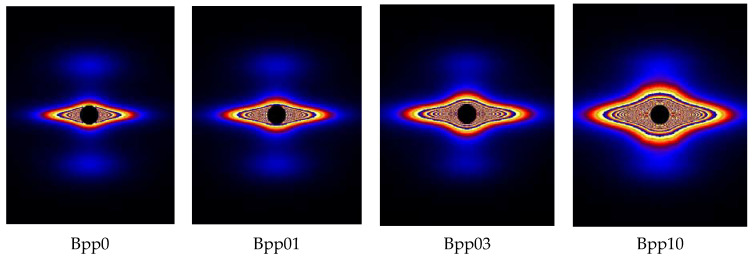
SAXS patterns of iPP with different amount of β-nucleating agents at strain of 300%. Both injection and stretching directions are vertical, linear-scale intensity.

**Figure 7:**
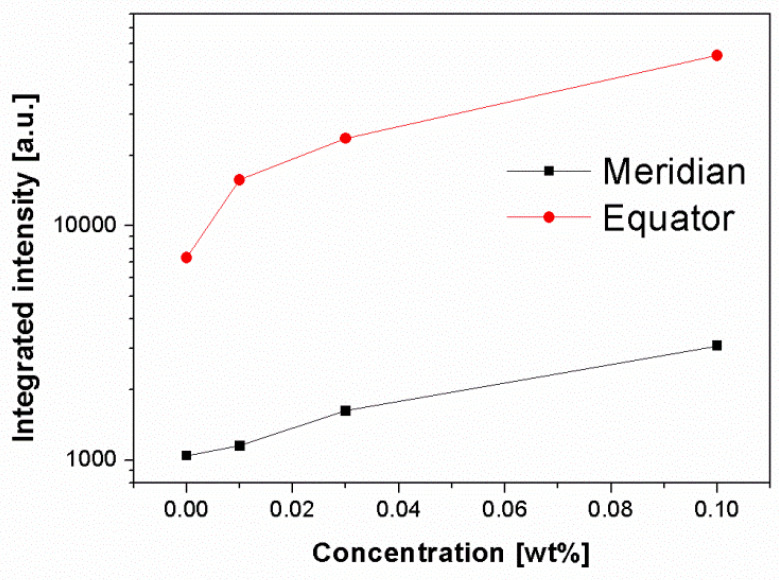
Integrated intensity along meridian and equator (same data processing as above) for deformed injection-molded iPP with strain of 300% as a function of β-nucleating agent concentrations.

Figure 6 shows the 2D-SAXS patterns of iPP samples nucleated with various concentrations of β-nucleating agent stretched to strain of about 300%. It can be observed that with increasing concentration of β-nucleating agent, the scattering intensity tended to increase. This can also be clearly seen in Figure 7. Combining with the strong equatorial streaks in the SAXS patterns, it can be inferred that the shape of voids and cavities was elongated along the stretching direction and their volume increased with increasing concentration of β-nucleating agent. To elucidate this phenomenon, the SAXS and WAXS data are further analyzed.

**Figure 8:**
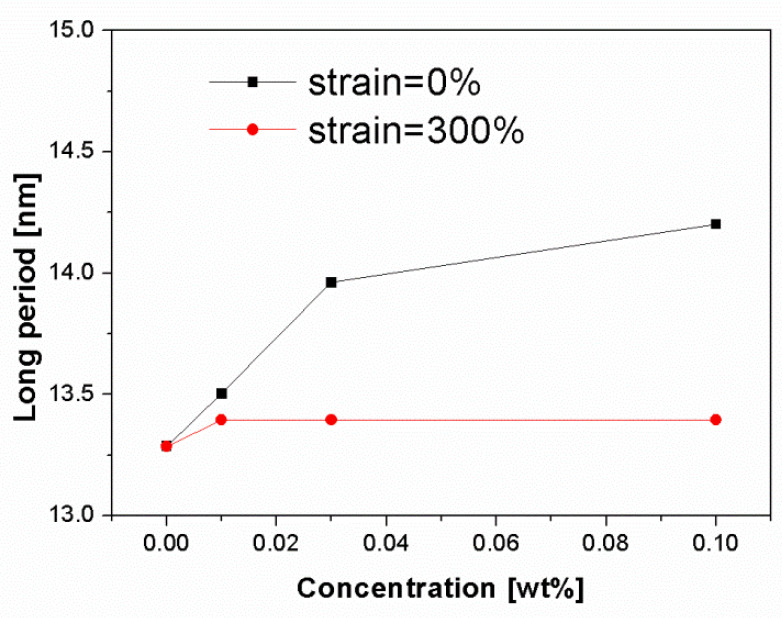
Change of long period of deformed and undeformed injection-molded samples as a function of β-nucleating agent concentrations, noting that the integral area was only selected at meridian to avoid the strong interference of cavities at equator.

Figure 8 shows the change of long period of deformed and undeformed injection-molded samples as a function of β-nucleating agent concentrations. It can be seen that the long period increased with increasing NJS concentration for undeformed samples, ranging from about 13.3 nm to 14.3 nm. As discussed above, the increased long period can be mainly attributed to the following two reasons: 1, high molecular alignment in shear region promoted by NJS particles; 2, increased β-content in core region with relatively thick lamellae thickness compared to α-lamellae. After stretching to 300%, the long period of β-nucleated iPP samples decreases to a constant value of 13.3 nm. This indicates that the deformation process promotes the fragmentation and rearrangement of crystallites followed the formation of thinner lamellae [23]. In addition, it is expected that the destruction of the thicker lamellae, which are more stable, may need higher stress, and thus, higher stress concentration was generated around the lamellae leading to formation of more voids and cavities.

**Figure 9:**
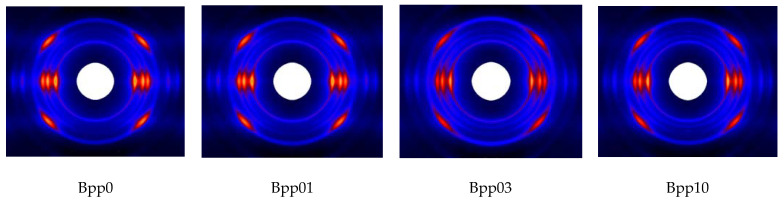
WAXS patterns of iPP with different amounts of β-nucleating agents at strain of 300%.

**Figure 10:**
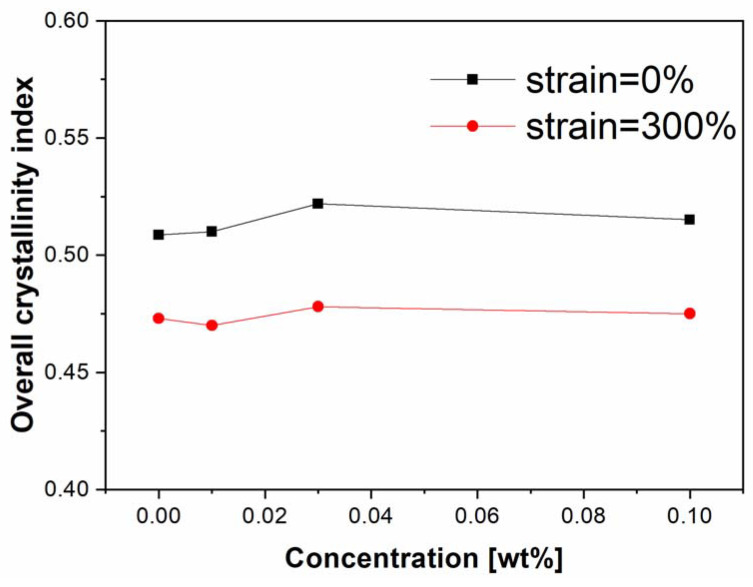
Overall crystallinity indices of deformed and undeformed injection-molded iPP samples as a function of β-nucleating agent concentrations.

Figure 9 displays the 2D-WAXS patterns of iPP samples nucleated with various concentrations of β-nucleating agent stretched to strain of about 300%. It is seen that those patterns were more or less identical regardless of the presence of weak β-(3 0 0) reflection in Bpp03 and Bpp10. Figure 10 shows the corresponding overall crystallinity indices of those undeformed and deformed samples. It can be seen that the overall crystallinity of undeformed samples was more or less similar with values of around 0.53. After stretching to 300%, it decreased to a value of around 0.45, indicating that the destruction of crystals occurred during the deformation process. To further elucidate this phenomenon, individual crystallinity was analyzed as follows.

**Figure 11:**
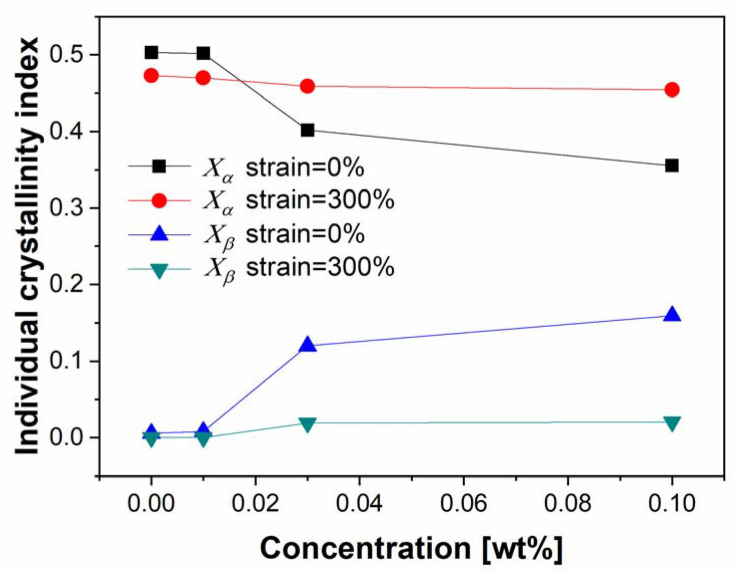
Individual crystallinity indices (*X_α_* and *X_β_*) of deformed and undeformed injection-molded iPP samples as a function of β-nucleating agent concentrations.

It can be seen in Figure 3 and Figure 9 that there are little if any (117) reflections in the 2D-WAXS patterns indicating the presence of few amount of γ-phase. The variation of γ-phase during deformation has been debated controversially. For example, Kalay et al. found that the γ-phase was stable and did not transform into α or any other phase during deformation [13], while Auriemma et al. pointed out that the γ form may gradually transform into α form upon stretching [50]. Since the scattering of γ-(117) reflection in our study is too weak to be further investigated, the following work will mainly concern the variation of β and α phases. Figure 11 shows individual crystallinity indices (*X_α_* and *X_β_*) of those iPP samples calculated based on 2D-WAXS patterns in Figure 3 and Figure 9. It is found that the β-crystallinity increased with increasing NJS concentration for the undeformed samples. After stretching to the strain of 300%, the β-crystallinity decreased in all of the samples. At the same time, the α-crystallinity increased for Bpp03 and Bpp10, indicating the occurrence of transformation from β- to α-crystals [51,52]. However, α-crystallinity decreased for Bpp0 and Bpp01. Noting that the original β-crystallinity for the undeformed Bpp0 and Bpp01 is too small (<0.01), the above-mentioned overall crystal destruction was mainly associated with the destruction of the α-crystals in these two samples. Still, it is reasonable to infer that such β-α transformation may take place during the deformation process of Bpp0 and Bpp01. In addition, it seems that such β-α transformation in Bpp10 with higher β-content was more pronounced than that in Bpp03 with relatively lower β-crystallinity. It is known that there is a density difference between α- and β-crystals with the density of α-crystals being higher than that of β-crystals [53]. Upon deformation, volume contraction is a nature result of β to α transformation [54]. The above increased volume of void and cavity is thus possibly associated with the β to α phase transition mainly occurred at the core region.

**Figure 12:**
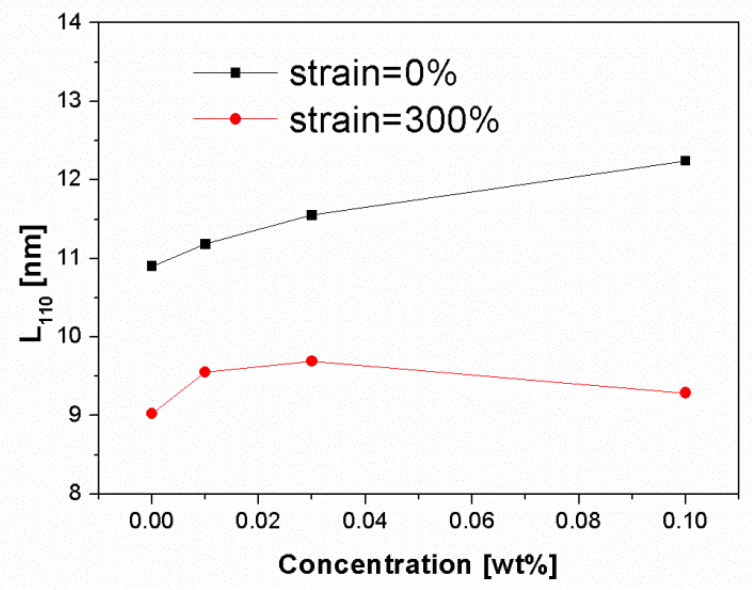
Crystallite size in the normal direction of the (1 1 0) reflection plane of deformed and undeformed injection-molded iPP samples as a function of β-nucleating agent concentrations.

Figure 12 shows the change of crystal size for those deformed and undeformed iPP samples. It can be seen that the crystal size increased with increasing NJS concentration for the undeformed samples. The addition of NJS may raise the crystallization temperature leading to the formation of stable and large crystals. After stretching to 300%, the crystal size markedly decreased, indicating that destruction of crystals occurred followed by the formation of smaller crystals during the deformation process.

**Figure 13:**
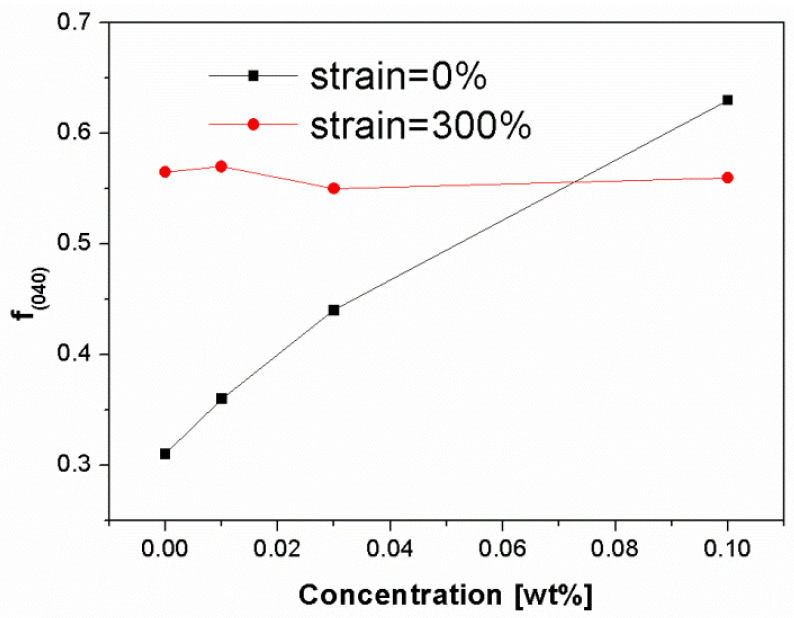
Orientation parameter of the lattice plane (0 4 0) for deformed and undeformed injection-molded iPP samples as a function of β-nucleating agent concentrations.

Figure 13 shows the change of orientation degree, based on the α-(0 4 0) lattice plane, of those deformed and undeformed iPP samples. It is found that the orientation degree increased with increasing NJS concentration for the undeformed iPP samples, ranging from 0.31 to 0.64. The orientation degree of sample was averaged from core to skin. As discussed above, highly oriented α-crystals are dominant in the shear layer of all the iPP samples. However, the core region of pure iPP and Bpp01 is mainly composed of α-crystals with little orientation which results in the decrease of their overall orientation degree. By contrast, with increasing NJS concentration (Bpp03 and Bpp10), the core region is gradually occupied by the β-crystals (Figure 2c) instead of α-crystals (Figure 2b). Thus the overall orientation degree is less influenced by the few α-crystals with little orientation in the core region and may keep at relatively high level. Furthermore, the flow intensity in shear layer may be strongly increased due to the interaction between flow and nucleating agent particles [55,56] leading to the more pronounced molecular alignment as well as the further increase of overall orientation degree. After stretching to 300%, all samples exhibit similar orientation degree with values of around 0.55. The increase of orientation degree in Bpp0, Bpp01 and Bpp03 may be attributed to the alignment of crystals including the original and new formed small crystals to the stretching direction, while the slight decrease of orientation degree in Bpp10 may be mainly associated with the destruction and rearrangement of crystals in shear layer as well as the formation of more α-crystals with possibly relatively less orientation degree in the core region due to the β to α phase transition as shown in Figure 11.

## 4. Conclusions

The synchrotron X-ray microbeam was used to identify the overall morphological distributions of injection-molded β-nucleated iPP, including distributions of crystallinity, long period and orientation, etc. It was found that the outer region (skin and shear region) of injection-molded iPP with relatively high concentration of NJS (i.e., ≥0.03 wt.%) was dominated by mainly highly oriented α-phase and certain amount γ-phase, while the core layer was rich in β-crystals with little if any orientation. The presence of nucleating agent particles promoted the alignment of molecular chains and induced more oriented nuclei, which was beneficial for the formation of lamellae with increased stacks distance in the shear layer. The long period increased with increasing concentration of β-nucleating agent in the core layer, since the β-nucleating agent may promote the formation of β-crystals with relatively large lamellar stacking distance.

Mini-dumbbell samples, of which the center coincided with the scanned position of the above morphology-identified samples, were further investigated by the in-situ synchrotron X-ray measurements coupled with mechanical testing to follow the structure variations upon deformation at strain of around 300%. It was found that voids and cavities along the stretching direction existed in the deformed iPP samples and their volume increased with increasing concentration of β-nucleating agent. The increased volume of void and cavity was associated with the β to α phase transition, which mainly occurred at the core region. The higher the concentration of β-nucleating agent used, the more the β-crystals transformed into the α-crystals during the deformation, thus the more the cavities were generated due to the density difference between α- and β-crystals. Upon deformation the fragmentation and rearrangement of crystallites occurred following the formation of thinner lamellae.

## Data Availability

Not applicable.
